# Irritable Bowel Syndrome: A Multifaceted World Still to Discover

**DOI:** 10.3390/jcm11144103

**Published:** 2022-07-15

**Authors:** Gabrio Bassotti

**Affiliations:** 1 Gastroenterology and Hepatology Section, Department of Medicine and Surgery, University of Perugia, 06131 Perugia, Italy; gabassot@tin.it; 2 Gastroenterology Unit, Perugia General Hospital, 06156 Perugia, Italy

Irritable bowel syndrome (IBS) is considered the prototype of disorders of gut–brain interaction (DGBI), and it is defined, according to Rome IV criteria, by the presence of abdominal pain or discomfort associated with bowel movements or changes in bowel habits with features of abnormal defecation. Notwithstanding an enormous amount of publications available in the literature, and the noticeable progress made by researchers in this field, IBS remains an enigma in several aspects, especially considering its pathophysiological grounds [1]. However, there are some well-established facts in this context, such as the underlying presence of abnormal gut–brain communication that, in turn, leads to visceral hypersensitivity, altered gut motility [2], and anomalous signal processing by the central nervous system [1].

An important point to consider is that IBS is also a multidimensional disorder, and it is frequently associated not only with other DGBI but also functional conditions of other origins (rheumatologic, urinary, etc.) [3]. This syndrome, still characterized by the absence of biological markers, plays an important role in the differential diagnosis of other gastrointestinal diseases, especially those with organic grounds. Therefore, investigating IBS patients is often quite complex (and sometimes frustrating) due to the multiple associations that can “muddy” the clinical picture [4,5]. Thus, after focusing for a long time on the motility/sensitive aspects of the gut, the research on IBS patients expanded to other areas, such as the presence of low-grade visceral inflammation and the relationships with other entities, making the IBS an increasing complex syndrome with several pathological overlaps. Moreover, the frequent association with psychologic/psychiatric abnormalities and with an impaired quality of life (QOL) characterizes IBS, and its treatment involves the use of multiple drugs. These above points have been taken into consideration by the studies presented in this Special Issue, which is dedicated to the latest research in IBS.

In a large retrospective study from Korea, involving more than 1,300,000 subjects, the authors investigated the role of medications used to treat IBS on the risk of osteoporosis and osteoporotic fractures [6]. Four groups were investigated: IBS patients, non-IBS patients, non-IBS patients using IBS drugs, and non-IBS patients not using IBS drugs. The drug categories taken into consideration were those to treat constipation, diarrhea, pain, and microbiota. After analyzing the data, the authors concluded that osteoporosis and osteoporotic fractures were significantly increased in subjects diagnosed with IBS that were using medications to treat the condition. Interestingly, this increase was also seen in subjects without IBS but who used drugs for the treatment of the condition. Notwithstanding the limits of a retrospective study and the possible occurrence of unidentified confounding factors, the authors concluded that patients with IBS (in whom the drug prescription rate is up to 80%), and those using medications to treat IBS symptoms, have an increased risk of osteoporosis and osteoporotic fractures. This observation is of clinical interest, raises some clinical concerns, and should be taken into consideration during follow-up for patients in longstanding treatment with these drugs.

In recent years, there had been a discrete interest in targeting the intestinal microbiota in IBS patients, since this target might be of potential therapeutic interest [7] (as also shown by experimental animal studies [8]) and affect comorbidities and QOL. Overall, the currently available results are conflicting, even though there is modest evidence of clinical improvement for some symptoms, at least in subgroups of IBS patients. This is due to several factors, one of the main factors being related to the fact that probiotics are not marketed as drugs: therefore, trials conducted with these agents do not match the standards imposed by regulatory agencies. In addition, there is the problem represented by the fact that the many different formulations available on the market are likely to have different effects. However, other IBS-related aspects have been scarcely investigated. In this Special Issue, authors from Germany conducted a systematic review with meta-analysis to assess the available evidence on the effects of probiotics on QOL and psychiatric symptoms (anxiety and depression) in IBS patients [9]. A data analysis, obtained in 11 out of 35 placebo-controlled studies, revealed that the QOL of IBS patients was similarly improved by placebo and probiotics, with a slight superiority for probiotics. The latter, however, had no effect on anxiety and depression. No symptom changes were detected between probiotics and placebo. The authors also recognized that there were limiting factors for this study, represented by the high heterogeneity of the trials (especially those conducted with single-strain probiotics) and the fact that QOL was usually reported as a secondary outcome in these trials, utilizing different assessment instruments. Thus, on the above basis, the authors concluded that a slight improvement documented in patients after probiotic treatment is unlikely to have a significant clinical relevance, and further and better-planned investigations are still needed. It also remains unknown whether there could be some benefit for IBS patients utilizing so-called psychobiotics, e.g., probiotics specifically developed in order to improve central nervous system functions [10]. 

Due to the fact that IBS is a frequent clinical condition and that its protean manifestations can overlap with several gastrointestinal pathological conditions, over the course of time, researchers’ attention also focused on the possible relationship between IBS and cancer. To date, this association is still widely debated, and no firm conclusions have been reached. In a study from Germany, included in this Special Issue, the incidence rates of different neoplasms were retrospectively assessed in two large cohorts of outpatients with or without IBS (about 20,000 subjects for each cohort were investigated) [11]. After analyzing the data, the authors concluded that IBS might be associated with digestive or extra-digestive neoplasms, with the greater incidence of cancers increasing in the first months after IBS was diagnosed. This suggests that, at least in some cases, symptoms of gastrointestinal cancer were falsely interpreted as being related to IBS, turning out to be untrue at the actual diagnosis after a short follow-up period. Although the study may have been biased by confounding factors, as also pointed out by the authors, such an observation should at the very least raise the alarm for a better investigation of seemingly innocuous gastrointestinal symptoms.

It is now well-established that the DGBI are frequently associated with fibromyalgia (FM). This association, focusing on IBS, has been investigated in this Special Issue by a group of Italian researchers, using the Rome IV criteria for the first time [12]. An analysis of the data confirmed precedent literature findings, and showed that almost 50% of FM patients had overlapping IBS (the most frequent condition found among the DGBI). Of these, the more numerous subtype was represented by the IBS m (mixed). Although limited by the low number of patients evaluated, and the fact that these were quite selective types being investigated in tertiary care institutions, the study enforces the concept that both IBS and FM likely share some common pathophysiologic grounds. These commonalities are probably represented by changes in the nociceptive system leading to increased pain thresholds, low-grade inflammation or other factors that can affect pain perception.

Since experts in the field usually formulate guidelines for IBS, there is the possibility of a translational gap between academic and practical daily medicine. This aspect was investigated by a group of Italian researchers, looking at the awareness and use of criteria to diagnose and treat IBS patients among a group of more than 200 general practitioners (GP) [13]. By considering the results obtained in this survey, slightly more than 50% of these GPs reported a satisfactory knowledge of IBS, although less than 40% considered that a professional training on IBS was useful to improve the awareness of this condition. About half of the GPs, and especially those that were younger, also claimed to be knowledgeable of Rome IV criteria for IBS diagnosis. Approximately the same percentage of GP reported knowledge and use in clinical practice of the Bristol Scale Stool Form and enforced the belief that additional symptoms (especially bloating) should be added to the diagnostic criteria for IBS. Concerning the clinical criteria used by GPs to diagnose IBS, the most frequent were represented by defecation-related abdominal pain, bloating, and variation in the number of defecations, with bloating considered as the most annoying symptom. By considering the pathophysiological grounds of IBS, the responses highlighted a wide range of uncertainty (abnormal gut motility, psychological triggering factors, gastrointestinal infections, gut dysbiosis, visceral hypersensitivity), with food intolerance/uncertainty surprisingly considered as a minor or less frequent cause for the patients’ complaints. Less than 50% of GPs reported achieving satisfying treatments for their IBS patients. Overall, notwithstanding the limiting factors (number of participants, male preponderance, prevalence of senior GPs), this study revealed the need for a constant update for GPs regarding this topic, taking into consideration the possibility of drawing shared guidelines for an easier clinical application. The fact that the mean age of GPs is 52 years also introduced another factor that is likely responsible for a translational gap and delayed updates on IBS.

In conclusion, it is increasingly more evident that IBS represents a wider world than previously expected, with multi-faceted aspects and relationships linking it to numerous other pathological conditions, which may sometimes be very challenging. Indeed, these aspects may strongly influence the clinical picture and modify therapeutic approaches, which remain quite unsatisfactory.
